# Total flavonoid contents in bamboo diets and reproductive hormones in captive pandas: exploring the potential effects on the female giant panda (*Ailuropoda melanoleuca*)

**DOI:** 10.1093/conphys/coy068

**Published:** 2019-04-10

**Authors:** He Liu, Chenglin Zhang, Yan Liu, Hejun Duan

**Affiliations:** 1 Beijing Key Laboratory of Captive Wildlife Technology, Beijing Zoo, No.137 Xi Zhi Men Wai street, Xicheng district, Beijing, China; 2 Beijing Municipal Key Laboratory of Food Poisoning Diagnosis Traceability Technology, Beijing Center for Disease Control and Prevention, No.13 He Ping Li Middle street,Dongcheng district, Beijing, China

**Keywords:** Diet bamboo, giant panda, phytoestrogen, reproductive hormone, total flavonoids

## Abstract

Phytoestrogens have been shown to affect the reproductive hormone levels in both humans and animals. As the main category of phytoestrogens, total flavonoids have a particularly important impact on female animals. To investigate the potential relationship between the total flavonoids in bamboo and the reproductive hormones in female giant pandas, urinary samples and dietary bamboo samples were collected from three main breeding locations (Beijing, Shaanxi and Sichuan). The chemical constituents of the total flavonoids in the bamboo were analysed and quantified using high-performance liquid chromatography coupled with a diode array detector (HPLC–DAD). Estradiol (E_2_), progestin (P), testosterone (T), luteinizing hormone (LH), follicle-stimulating hormone (FSH) and prolactin (PRL) were measured via radioimmunoassay (RIA). The results revealed that the total flavonoids in the bamboo from Sichuan were significantly higher than those in the bamboo from Beijing and Shaanxi, and the concentration in bamboo from Shaanxi was higher than that from Beijing (*P* < 0.05). The urinary E_2_, P, T, FSH and LH levels in pandas from Beijing were significantly lower than those in pandas from Sichuan and Shaanxi (*P* < 0.05). The concentrations of six reproductive hormones were positively associated with the total flavonoid contents in bamboo. In addition, the birth rate of pandas in Sichuan was significantly higher than the birth rate of pandas in Beijing and Shaanxi (*P* < 0.05). Thus, the flavonoids of bamboo may be related to reproduction and giant pandas might retain a sensitive adaptation to phytoestrogens from bamboo. The total flavonoids of bamboo may play a distinct role in the reproductive success of giant pandas.

## Teaser

This research illustrates the relationship between the total flavonoids content in bamboo diets and the reproductive hormones of female giant pandas from three locations with different birth rates. The flavonoids of bamboo may be related to reproduction and giant pandas might retain a sensitive adaptation to phytoestrogens from bamboo.

## Introduction

Phytoestrogens are plant-derived oestrogens that are not generated within the endocrine system but are consumed only by eating phytoestrogenic plants (Bennett *et al.*, 1946; Dusza *et al.*, 2006). Hundreds of plants have been found to contain phytoestrogens, including some well-known isoflavonoids, stilbenes, lignans and coumestans, and these compounds can bind to oestrogen receptors and mimic the action of oestrogens on target organs and cause oestrogenic effects on animals and humans (Dusza *et al.*, 2006; Mlynarczuk *et al.*, 2011). Phytoestrogens are not considered nutrients because they do not participate in any essential biological function (Chen, 2004). However, phytoestrogens have been found to promote growth, improve immunity and lactation performance and even affect the egg production performance of animals (Wang *et al.*, 2013).

Many specific studies have demonstrated the various impacts of phytoestrogens on reproduction in birds and mammals, and these studies showed that phytoestrogens act variably when they are consumed by different species and genders and administered through different routes in captive animals (Murkies *et al.*, 1998; Setchell and Cassidy, 1999; Ball *et al.*, 2010; Lu *et al.*, 2010; Wasserman *et al.*, 2012a,b; WocBawek-Potocka *et al.*, 2013; Sittipon *et al.*, 2014). Still, the effect of food supplementation in long-term on populations remains unknown and few are published on the research of wildlife (Birnie-Gauvin *et al.* 2017). Since bamboo accounts for 99% of the diet of giant pandas (*Ailuropoda melanoleuca*) (Schaller *et al.*, 1985), phytoestrogens may have a substantial influence. However, the impact of total flavonoids in zoo-housed giant pandas has not been reported. To obtain a better understanding of the reproductive process of these animals, the potential relationship between total flavonoids in bamboo diets and the reproductive hormones of giant pandas must be investigated.

Giant pandas feed on a wide variety of bamboo within various localities in China (Zhou and Huang, 2005; Fu *et al.*, 2008). In the Qinling Mountain area of Shaanxi Province, 17 species of bamboo are consumed by giant pandas, including *Fargesia denudata, Fargesia rufa* and *Fargesia dracocephala* (Tian, 1989; Huang, 1995; Li *et al.*, 2003; Mo *et al.*, 2004). In Sichuan Province, giant pandas prefer to feed on eight species, including *Yushania lineolata*, *Bashania fangina*, and *Bashania spanostachya* (Fu *et al.*, 1988; Qin *et al.*, 1993; Zhou *et al.*, 1996). Several studies have indicated that different bamboos contain different flavonoid constituents, such as orientin, isoorientin, isovitexin, vitexin and tricin (Wei *et al.*, 2013; Guo *et al.*, 2014; Pan *et al.*, 2014). Even if the same flavonoids are present, the concentration levels will vary significantly among different species of bamboo. For example, the maximum level of flavone C-glycosides was found in *Phyllostachys nigra*, while the minimum level was found in *Phyllostachys propinqua* (Li *et al.*, 2004). Additionally, the diversity of flavonoid content has been shown to vary based on the geographic distribution of bamboo; the flavonoid level from the Meishan area was the highest and the level in the Yaan area was comparatively lower (Yu *et al.*, 2008).

Giant pandas housed in Chinese zoos are traditionally fed different bamboo species. There are three main captive populations of giant pandas. Giant pandas from Shaanxi Province are primarily fed bamboo from the *Phyllostachys* genus, whereas those from Sichuan Province are fed *Pleidolaslus amarus* and *Phyllostachys bissetii*, and those from Beijing are fed *P. propinqua*. Sichuan and Shaanxi have a significant history of suitable reproductive performance, whereas Beijing presents lower reproductive performance based on a scientific comparison (Xie, 2014).

Female giant pandas from Beijing were moved to Sichuan for breeding since 2011, and ten cubs have been born in Sichuan during 2012 to 2018. Similar husbandry and management were implemented at these three institutes; thus, the question remains as to whether the bamboo diets of these pandas played an important role in their reproductive success. Giant pandas usually have a delayed implantation during the reproduction cycle, so the reproductive hormone are basically at a very low level during the oestrus cycle from March to May (Lindburg *et al.*, 2001; Zhang and Wang, 2003; Zhang *et al.*, 2009). Further, the urine estradiol (E_2_) level closely correlates to the estrus of giant pandas (Zeng *et al.*, 1994, McGeehan *et al.*, 2002). The objective of this study is to determine whether differences are observed in reproductive hormones levels based on the consumption of different bamboos and to investigate the potential influence of bamboo phytoestrogens on the reproduction of giant pandas.

Phytoestrogens may vary according to bamboo species and distribution (Li *et al.*, 2004; Yu *et al.*, 2008), and their contents may affect the hormone levels of giant pandas. We examined the concentrations of total flavonoids of dietary bamboo from three collections as well as the urinary hormone levels of E_2_, progestin (P), testosterone (T), follicle-stimulating hormone (FSH), luteinizing hormone (LH) and prolactin (PRL) among giant pandas in three locations. Specifically, this study aims to determine whether the total flavonoids in different species of bamboos are potentially related to urinary hormone levels in adult female giant pandas.

## Material and methods

### Animal and bamboo diets

The animals in this study included 11 female giant pandas at three sampling locations (Table 1), and the samples were collected during the reproductive season (from March to May). At the same period of time, urinary samples from the giant pandas and their bamboo diets were simultaneously collected by random sampling. Three pandas in this sampling were from Beijing Zoo, four were from the Sichuan Wolong Nature Reserve, and four were from the Shaanxi Rare Wild Animals Rescue and Breeding Research Centre. The main species of bamboo fed to the giant pandas were as follows: *P. propinqua McClure* from Beijing, *P. amarus* from Sichuan and *Phyllostachys aureosulcata Spectabilis* from Shaanxi. Approximately 2–3 kg bamboos for each species were randomly collected from three locations and stored at the low temperature. In accordance with the technical regulations of husbandry and management of the giant pandas published by China standard press (2012), the daily diets in three locations consisted of 75–90% fresh bamboo and 10–25% supplemental feed, which included steamed bread, apples and carrots.

**Table 1:** The details for bamboo samples, urinary samples and female giant pandas

Giant pandas	Birth year	Collections	Urinary sample	Bamboos	Breeding time (years)	Bamboo samples
Yinghua	2003	Beijing	10	*Phyllostachys propinqua McClure*	5	20 pieces
Mengmeng	2006	Beijing	10		3	
Jini	1993	Beijing	10		10	
Haizi	1994	Sichuan	12	*Pleidolaslus amurus*	8	20 pieces
Long Xin	2000	Sichuan	12		6	
Zizhu	2002	Sichuan	12		4	
Guoguo	1996	Sichuan	12		7	
Xinxin	2005	Shaanxi	11	*Phyllostachys aureosulcata Spectabilis*	3	20 pieces
Yangyang	2003	Shaanxi	11		5	
Zhuzhu	2000	Shaanxi	11		5	
Niuniu	1997	Shaanxi	11		7	

### Sample collection

The giant pandas usually eat not only the culms but also the leaves of bamboo from March to May. Fresh whole bamboo diets were collected by random sampling at the three zoos from the same time period of 2014. *P. propinqua McClure* from Beijing, *P. amarus* from Sichuan, *P. aureosulcata Spectabilis* from Shaanxi were the main categories of food. All collected bamboos from the three locations were shipped to a Beijing laboratory and stored at 4°C until further analysis. Urinary samples from the 11 female giant pandas were collected at the three locations. Urinary samples were collected from the indoor floor between 06:00 and 11:00 by a syringe and labelled in cryotubes following sampling (Lindburg *et al.*, 2001). The keepers took the sample usually <2 h between urination and collection. Approximately 10 ml of urine was collected daily while avoiding contamination from faeces and food over a 12-day period. Urinary samples from the three locations were stored cold and express shipped to the Beijing laboratory, where they were stored at −80°C until subsequent analysis.

### Determination of the total flavonoid contents from the bamboo diets

The total flavonoids of these bamboo diets were determined and quantified using a high-performance liquid chromatography coupled with a diode array detector (HPLC–UV/DAD) system (Waters Co. Ltd, USA). Whole bamboo samples were dried in an oven at 40 ± 2°C for 48 h and triturated in an RRH-A400 pulverizer (Zhejiang Province, China). The samples were ground at 800 r/min for 6 min to obtain a fine powder. All samples were then bagged and stored in a desiccator until further analysis.

The analytical standard rutin (quercetin-3-*O*-rutinoside, 98% purity) was purchased from Sigma-Aldrich (China). Analytical grade ethyl alcohol, sodium carbonate, sodium bicarbonate, and phosphoric acid were purchased from the Beijing Chemical Reagent Company (Beijing, China). Methanol and acetonitrile (HPLC grade) were provided by Dikma Co., Ltd (Beijing, China).

Fine bamboo powder (2.0 g) was immersed in 80 ml 75% (v/v) aqueous ethanol for 24 h and decoloured by adding ~2 g active carbon over one hour. After centrifuging for 5 min, the supernatant was concentrated using rotary evaporation to remove the ethanol. Finally, Millipore-filtered water was added to each sample tube to yield a final volume of 20 ml for analysis. A HPLC-UV/DAD system (Waters, USA) coupled with a C18 column 4.6 mm × 250 mm (Agilent, USA), was used to determine the total flavonoids. Mobile phase A was set by acetonitrile, and the composition of mobile phase B was set by phosphoric acid buffer (pH = 2.8) in water. The isocratic elution using A/B (45:55, v/v) was applied at a total flow rate of 1.0 ml/min. The absorbance of the signal using a diode array detector was 355 nm.

### Radioimmunoassay determination of urinary hormones

The urinary hormones E_2_, P, T, FSH, LH and PRL were obtained from the three giant panda locations (Beijing, Shaanxi and Sichuan) and analysed using E_2_, P, T, FSH, LH and PRL radioimmunoassay (RIA) kits from Beijing North Institute of Biological Technology (Beijing, China). The urinary hormone concentrations were measured by a 125 I-based RIA. The urine samples were corrected by the creatinine value to control the variability in the urine concentration. The units of hormonal values were transformed to mass units per milligrams of creatinine (Taussky,1954). These assays were performed base on the manufacturers’ recommendations for urine samples. All samples were analysed simultaneously using the same microtiter plate to decrease unexpected variations (Cekan, 1975). Recovery of known amounts of the six hormones respectively added to a pool of diluted panda urine (50 μl, 1:5) ranged 78–103% (*n* = 6), which met the test requirement. The liners of six hormones added in diluted urine samples showed a good relationship (*r* = 0.98–0.99). The intra-assay and inter-assay coefficients of variations were <10 and 15%, respectively. Hormonal values under 0.1 mg ml^−^^1^ were considered below the range of sensitivity and replaced by the limit of detection. Undetected values accounted for <5% of the total measurements.

### Statistical analysis

The resulting hormone values were transformed into mass units per milligrams. The equation for calibration transformations is 1 μmoL l^−1^ = (8840 mg ml^−1^)^−1^. The annual birth rate was determined by the number of newborns among the total number of animals in a breeding area in a year. The average annual birth rates of three locations were calculated according to the studbook from 2002 to 2014 (Xie, 2014).

The Kolmogorov–Smirnov test was applied to determine whether the dataset matched the assumption of normality. After its application, the proper statistical tests had been chosen on the different datasets. The Kruskal–Wallis *H* test as nonparametric test was used to identify the hormone differences among the three captive groups. The Mann–Whitney test was used to compare the differences in bamboo flavonoids between two collections. Spearman tests were applied to determine the significance of the correlations between the hormone parameters and total bamboo flavonoids. One-way ANOVA as parametric test was used to analyse the birth rate differences in three locations. The homogeneity of variance was analysed using Levene’s test, and multiple comparisons within groups were performed using post hoc tests.

SPSS 17.0 for Windows (SPSS Inc., Chicago, IL, USA) was used for the statistical analyses. Differences were considered significant at *P* < 0.05. The data were described as the mean ± SE.

## Results

### Total flavonoid contents in the bamboo diets

**Figure 1:**
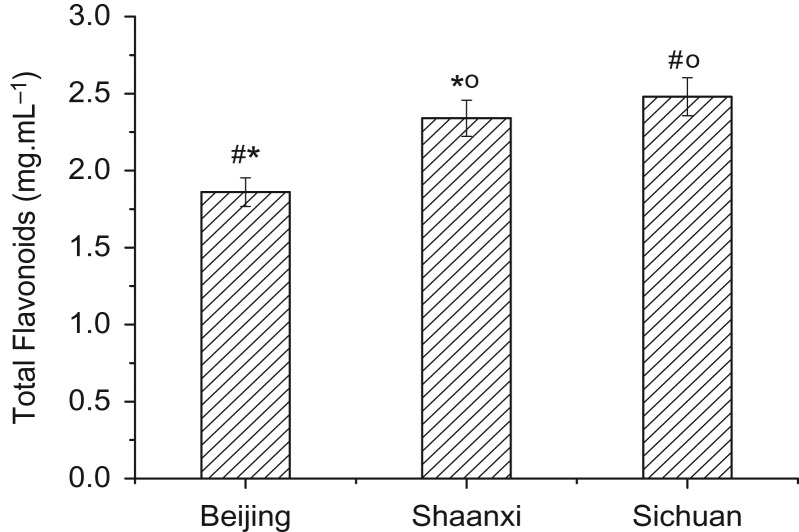
The Concentration of total flavonoids in different bamboos at three giant panda collections. ‘#’ indicates significant difference between Beijing and Sichuan; ‘o’ indicates significant difference between Beijing and Shaanxi, ‘*’ indicates significant difference Shaanxi and Sichuan. All significance is *P* < 0.05

The total flavonoid content in the bamboo diets of pandas from Sichuan was significantly higher than that in the bamboo diets of pandas from Beijing (Mann–Whitney test *Z* = −2.121, *P* = 0.034; Fig. 1). Additionally, the total flavonoid content in the bamboo from Shaanxi was lower than that in the bamboo from Sichuan (*Z *= −2.309, *P =* 0.021; Fig. 1) and statistically higher than that in the bamboo from Beijing (*Z* = −2.121, *P* = 0.034; Fig. 1).

### Reproductive hormone levels in pandas at the three locations

**Figure 2:**
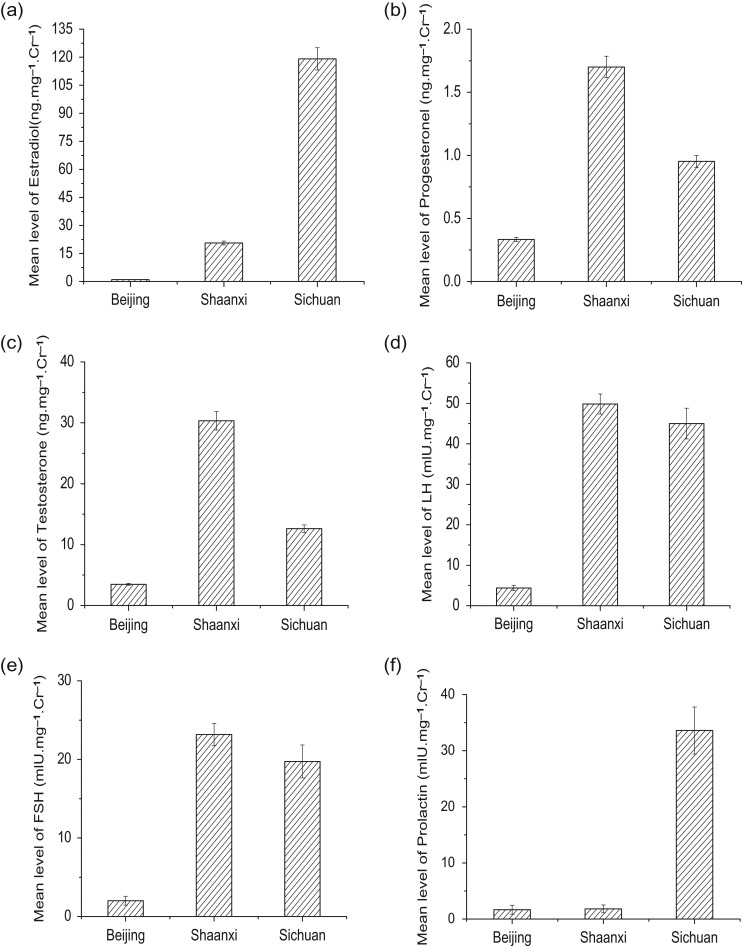
Urinary hormone levels of giant pandas in three collections. (**a**) Estradiol, (**b**) progesterone, (**c**) testosterone, (**d**) LH, (**e**) FSH and (**f**) PRL. Data are mean ± SE

The overall urinary E_2_, P, T, FSH and PRL levels were significantly different among the three locations, whereas statistically significant differences were not observed for LH (Kruskal–Wallis test, Table 2). The urinary E_2_ in Beijing zoo samples was significantly lower than that in the Shaanxi and Sichuan zoo samples (Mann–Whitney test, Table 2 and Fig. 2a). The urinary E_2_ from the Sichuan pandas was significantly higher than that from the Shaanxi pandas (Table 2 and Fig. 2a). However, the P and T of the Shaanxi zoo pandas were the highest among the three locations (Table 2 and Fig. 2b and c), whereas the P and T of the Beijing zoo pandas were significantly lower than those of the other panda groups (Table 2; *P* < 0.05, Fig. 2b and c).

**Table 2:** Analysing the six reproductive hormones of giant pandas at three collections

Item	Main effect			Group comparison		
	df	*X*^*2*^	*P*	Beijing vs. Shaanxi	Beijing vs. Sichuan	Shaanxi vs. Sichuan
E_2_ (ng mg^−1^)	2	8.909	0.012^*^	**+**	**+**	**+**
P (ng mg^−1^)	2	8.956	0.011^*^	**+**	**+**	**+**
T (ng mg^−^^1^)	2	8.227	0.016^*^	**+**	**+**	**+**
LH (mIU mg^−^^1^)	2	5.053	0.080	**−**	**+**	**−**
FSH (mIU mg^−^^1^)	2	7.136	0.028^*^	**+**	**+**	**−**
PRL (mIU mg^−^^1^)	2	7.053	0.029^*^	**−**	**+**	**+**

Kruskal–Wallis test was used to analyse main effect. Mann–Whitney test was performed between groups. The significant threshold value equals to 0.05. The result interprets: ‘+’ shows the significant value (*P* < 0.05), while ‘−’ presents the not significant value (*P* > 0.05).

Although the levels of LH did not show differences among the three locations (Kruskal–Wallis test, Table 2), the measurements of the Beijing animals differed from those of the Sichuan and Shaanxi animals (Mann–Whitney test, *P* < 0.05; Fig. 2d). FSH levels in the Beijing pandas were significantly lower than those in the Shaanxi or Sichuan pandas, but there were no differences in FSH between the Shaanxi and Sichuan pandas (Fig. 2e). Compared to the samples from Beijing and Shaanxi pandas, urinary PRL concentrations in the Sichuan pandas were significantly higher (*P* < 0.05; Fig. 2f). However, there was no significant difference in PRL samples between the samples from the Shaanxi and Beijing pandas (*P* > 0.05; Fig. 2f).

### Total flavonoid contents in the bamboo diets and reproductive hormone levels in the pandas

**Figure 3:**
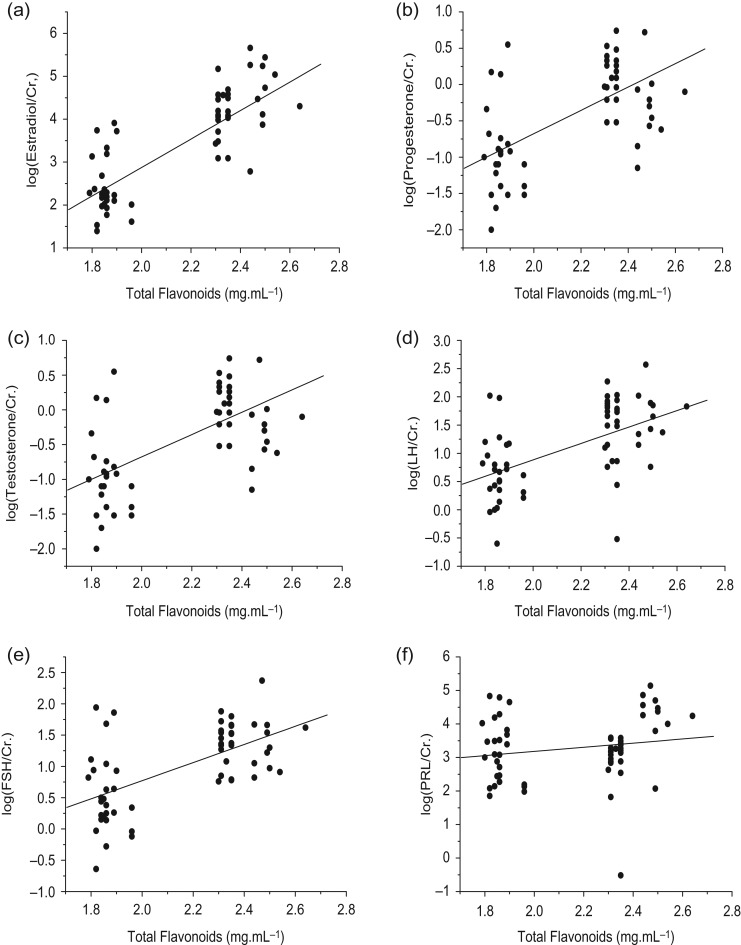
The relationship between total flavonoids of bamboo and urinary hormones in giant pandas. (**a**) Estradiol, (**b**) progesterone, (**c**) testosterone, (**d**) LH, (**e**) FSH, and (**f**) PRL. The hormone measurements were transformed by logarithm for statistics

To determine whether the total flavonoids of bamboo diets affected the level of reproductive hormones in female giant pandas, urinary samples and bamboo samples from the three study locations were analysed via Spearman’s correlation. All six reproductive hormones showed a positive correlation with the total flavonoids in the bamboo species. The urinary E_2_ was statistically correlated with the concentration of total flavonoids (*r* = 0.726, *P* < 0.01; Fig. 3a). Similar to urinary E_2_, the P (*r* = 0.490, *P* < 0.01; Fig. 3b), T (*r* = 0.468, *P* < 0.01; Fig. 3c), LH (*r* = 0.465, *P* < 0.01; Fig. 3d) and FSH (*r* = 0.503, *P* < 0.01; Fig. 3e) concentrations were significantly correlated with the total bamboo flavonoids, although urinary PRL was less strongly correlated with the total bamboo flavonoids (*r* = 0.279, *P* = 0.03, Fig. 3f).

### Birth rates at the three locations

**Figure 4:**
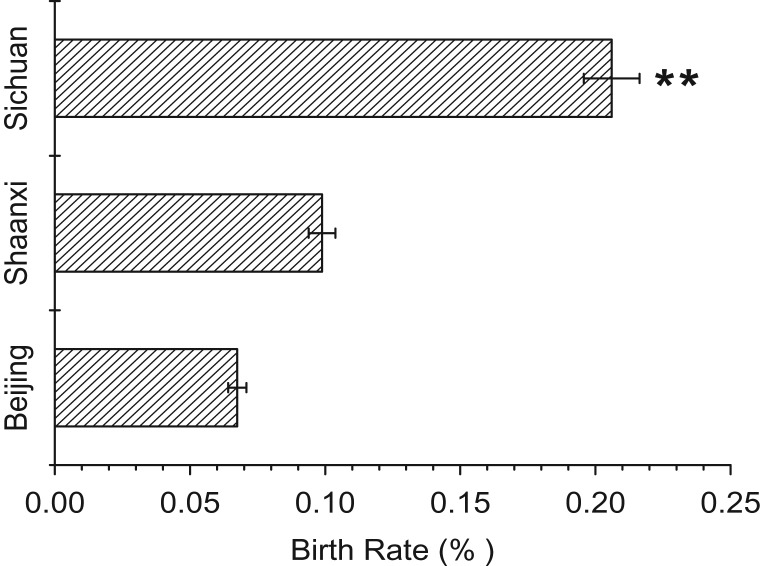
Birth rate of giant pandas from three locations: Beijing, Shaanxi and Sichuan. ** indicate statistically difference with the other two groups (*P* < 0.05)

Significant differences were observed among the three locations (one-way ANOVA *F* = 5.004, *P* = 0.015). The average annual birth rate in the Sichuan facility (20.60 ± 2.90) was significantly higher than that in the Beijing and Shaanxi facilities (*post hoc* tests; *P* = 0.006 and *P* = 0.028, respectively; Fig. 4). The average annual birth rate in the Beijing facility was lower than that in the Shaanxi facility (6.75 ± 3.01, 9.89 ± 3.72, respectively), although the difference was not statistically significant (*P* = 0.501; Fig. 4).

## Discussion

The composition of total flavonoids in a plant is generally dependent on the species, although it can be altered by environmental factors (Whitten *et al.*, 1995). Even in similar bamboo species, the concentration of total flavonoids varies according to different geographic distributions (Yu *et al.*, 2008). In this research, the concentrations of total flavonoids were different in the three locations. The total flavonoid content in *P. propinqua* from Beijing was lower than those in *P. amarus* from Sichuan as well as those in *P. aureosulcata* in Shaanxi. Our results were consistent with previous studies in which the total flavonoids of *P. propinqua* were the lowest among eight bamboo species (Li *et al.*, 2004).

According to the urinary hormone levels, we observed that the hormones (except for PRL) in the Beijing samples were lower than those in the Shaanxi or Sichuan samples. All reproductive hormones in the Sichuan samples were significantly higher than those in the Beijing samples. Additionally, the urinary E_2_ concentrations in the samples from Sichuan and Shaanxi were higher than those in the samples from Beijing. Many studies have shown that urinary E_2_ concentrations in giant pandas were lower during proestrus and then suddenly increased during oestrus, which indicates that the reproductive hormone status was a key factor in the pandas’ reproductive success (Shi *et al.*,1988; Li *et al.*, 1993; Xie *et al.*, 1993). Our findings revealed that urinary E_2_ concentrations remained at low levels in the Beijing pandas, which were primarily fed the bamboo species *P. propinqua*. Furthermore, the birth rate of giant pandas from the Beijing zoo experienced a slowdown over past decades (Xie, 2014). The female giant pandas of Beijing zoo have been bred and raised on a diet of *P. amarus* and *P. bissetii* at the Sichuan facility since 2011, the birth rate of the population has slightly increased in Beijing in recent years.

In our findings, the concentrations of six reproductive hormones were positively associated with the total flavonoid contents of bamboos. As observed in prior studies, wild giant pandas select different parts of multiple bamboo species throughout a year (Wei *et al.*, 1999; Hu *et al.*, 2000). One of the reasons for this variation might be seasonal fluctuations in the concentration of flavonoids in different bamboos (Su *et al.*, 2011). Moreover, the total flavonoid contents in various bamboos are higher in spring (Lü *et al.*, 2011; Pan *et al.*, 2014). Zhao *et al.* (2013) found that giant pandas preferred a slightly bitter bamboo species in which the bitter taste was primarily associated with certain flavonoids. Although the total flavonoids in the bamboo diets were only found in trace amounts, the target compositions could be accumulated *in vivo* due to daily consumption (Schaller *et al.*, 1985; Sarita *et al.*, 2008). Wynne-Edwards (2001) found that a lower concentration of phytoestrogens compared with endogenous oestrogens was still related to reproduction in domestic mammalian herbivores. The phytoestrogens of bamboo diets are therefore considered a main factor that influences giant pandas (Yuan *et al.*, 2015), and the concentrations of total flavonoids may have a close relationship with panda reproduction.

E_2_ is the foundation of oestrus in female mammals, playing an important role of beginning in reproduction (Wallen and Goy, 1977; Lipschitz, 1997). Little is known about the activity of phytoestrogens on reproduction in terrestrial mammals, although in recent years, phytoestrogens and their potential functions have gained considerable attention in scientific research (Emery Thompson *et al.*, 2008; Lu *et al.*, 2010; Wasserman *et al.*, 2012a,b). In particular, phytoestrogens may cause a significant oestrogenic or antioestrogenic effect that disrupts endocrine systems potentially, which results in chemical castration for domestic mammalian herbivores and affects reproduction (Wynne-Edwards, 2001; Dusza *et al.*, 2006; Ryökkynen, 2006; Rochester and Millam, 2009). The results of this study demonstrate that the total flavonoids of a bamboo diet are positively related to the reproductive hormone levels in giant pandas and that the total flavonoids from bamboo may contribute to oestrogenic achievement. As an herbivorous carnivore, giant pandas may retain a sensitive adaptation to phytoestrogens.

Previous studies reported that high levels of phytoestrogens were implicated in adverse effects on reproduction and led to reproductive failure in sheep, cattle, equol and rodents (Dusza *et al.*, 2006; Mlynarczuk and Kotwica, 2006). Moreover, the consumption of oestrogenic leaves was positively correlated to faecal E_2_ levels in red colobus (Wasserman *et al.*, 2012a). Our findings indicate that the total flavonoid content affects the reproduction of female giant pandas, and all six reproductive hormones were positively associated with flavonoid concentrations in bamboo. Thus, our results show that phytoestrogens from bamboo promote hormone levels in giant pandas. Specifically, urinary E_2_ levels are significantly linked to the concentration of bamboo flavonoids. Considering the similar outcomes for other reproductive hormones, the total flavonoids in a bamboo diet may play a distinctive role in regulating reproductive hormones in female giant pandas in captivity.

## Conclusions

Overall, this research illustrates the relationship between the total flavonoid content in bamboo diets and the reproductive hormones of female giant pandas from three locations with different birth rates in China. The reproductive hormone levels of giant pandas in Beijing were relatively low compared with the levels of pandas from Sichuan and Shaanxi, and smaller total flavonoid amounts were found in the bamboo species fed to Beijing pandas. The total flavonoids in various bamboos influence the reproductive hormones in zoo-housed giant pandas. These findings complement relevant studies on dietary phytoestrogens and highlight the potential effects of total flavonoids for increasing the hormone levels of E_2_ and P. Based on these findings, an improved dietary plan may be necessary for giant pandas in zoos. Further research is needed to clarify the specific mechanisms underlying the regulation of reproduction by phytoestrogens.
